# Supplementary material for: Characterization of the Mitochondrial Genome of the Vietnamese Central Highland Wild Boar (Sus scrofa)
Source: Animals (Basel). 2025 Jul 10;15(14):2029. doi: 10.3390/ani15142029 (PMC12291927; doi:10.3390/ani15142029)
Supplement: Supplementary file 1 [file animals-15-02029-s001.zip › Data S1.pdf]

Supplementary S1: Complete mtDNA genome of Vietnamese *Sus scrofa*

CGCATATAAGCAGGTAAATTATTAGCTCATTCAAACCCCCCTTACCCCCCATTAAACTTATGCT
CTACACACCCTATAACGCCTTGCCAAACCCCAAAAACAAAGCAGAGTGTACAAATACAATAA
GCCTAACTTACACTAAACAACATTTAACAACACAAACCACCATATCTTATAAAACACTTATAAAA
CACTTACTTAAATACGTGCTACGAAAGCAGGCACCTACCCCCCTAGATTTTTACGCCAATCTA
CCACAAATAAGTTTAAAATTACAACACAATAACCTCCCAAAATATAAGCACCTATTTAAGCATAC
GCCCACAATCTGAATATAGCTTATAGTTAATGTAGCTTAAATTATCAAAGCAAGGCACTGAAAA
TGCCTAGATGGGCCTCACAGCCCCATAAACACACAGGTTTGGTCCTGGCCTTTCTATTAATT
CTTAATAAAATTACACATGCAAGTATCCGCGCCCCGGTGAGAATGCCCTCCAGATCCTAAAG
ATCAAAAGGAGCAGGTATCAAGCACACCTATAACGGTAGCTCATAACGCCTTGCTCAACCAC
ACCCCCACGGGAAACAGCAGTGATAAAAATTAAGCCATGAACGAAAGTTTGACTAAGTTATAT
TAATTAGAGTTGGTAAATCTCGTGCCAGCCACCGCGGTCATACGATTAACCCAAATTAATAGA
TCCACGGCGTAAAGAGTGTTTAAGAAAAAAAAACCACAATAGAGTTAAATTATAACTAAGCTG
TAAAAAGCCCTAGTTAAAATAAAATAACCCACGAAAGTGACTCTAATAATCCTGACACACGATA
GCTAGGACCCAAACTGGGATTAGATACCCCACTATGCCTAGCCCTAAACCCAAATAGTTACAT
AACAAAACTATTCGCCAGAGTACTACTCGCAACTGCCTAAAACTCAAAGGACTTGGCGGTG
CTTCACATCCACCTAGAGGAGCCTGTTCTATAATCGATAAACCCCGATAGACCTTACCAACCC
TTGCCAATTCAGCCTATATACCGCCATCTTCAGCAAACCCTAAAAAGGAACAATAGTAAGCAC
AATCATAACACATAAAAACGTTAGGTCAAGGTGTAGCTTATGGGTTGGAAAGAAATGGGCTA
CATTTTCTACATAAGAATACCCACCATACGAAAGTTTTTATGAAACTAAAAACCAAAGGAGGAT
TTAGCAGTAAATCAAGAATAGAGTGCTTGATTGAATAAGGCCATGAAGCACGCACACACCGC
CCGTCACCCTCCTCAAGCATGTAGTAATAAAAATAACCTATATTCAATTACACAACCATGCAAG
AAGAGACAAGTCGTAACAAGGTAAGCATACTGGAAAGTGTGCTTGGATTACCAAAGCATAGC
TTAAACTAAAGCACCTAGTTTACACCTAGAAGATCCCATAATATATGGGTACTTTGAACCAAAG
CTAGCTCAACATATTAAACAAATACAAAAATACACCAAAATAAAATAAAACATTCACCTAACATT
AAAGTATAGGAGATAGAAATTTTTATCCTGACGCTATAGAGATAGTACCGTAAGGGAAAGATG
AAAGAATAAAATAAAAGTAAAAAAAAGCAAAGATTACCCCTTCTACCTTTTGCATAATGGTTTA
ACCAGAAAAAATCTAACAAAGAGAACTTTAGCTAGATACCCCGAAACCAGACGAGCTACCTA
TGAGCAGTTTAAAAGAACCAACTCATCTATGTGGCAAAATAGTGAGAAGACTTGTAGGTAGA
GGTGAAAAGCCTAACGAGCCTGGTGATAGCTGGTTGTCCGAGAAAGAATTTTAGTTCAACC
TTAAAAATACCCCAAAAACCCTAAATTCCAATGTATTTTTAAGAGATAGTCTAAAAAGGTACAG
CTTTTTAGAAACGGATACAACCTTGACTAGAGAGTAAAATCTTAATACTACCATAGTAGGCCTA
AAAGCAGCCATCAATTGAGAAAGCGTTAAAGCTCAACAAATTCACCAACATAATCCCAAAAA
CTAATAACAAACTCCTAGCCCAATACCGGACTAATCTATTGAAACATAGAAGCAATAATGTTAA
TATGAGTAACAAGAAGCCTTTCTCCTCGCACACGCTTACATCAGTAACTAATAATATACTGATA
ATTAACAATCAATAAACCAAAACAACACTAAAGCGTTTATTAATTATATTGTTAACCCAACACAG
GAGTGCACCAAGGAAAGATTAAAAGAAGTAAAAGGAACTCGGCAAACACAAACCCCGCCTG
TTTACCAAAAACATCACCTCTAGCATTACTAGTATTAGAGGCAATGCCTGCCCAGTGACACCA
GTTTAACGGCCGCGGTATTCTGACCGTGCAAAGGTAGCATAATCACTTGTTCTCCAAATAAG
GACTTGTATGAATGGCCACACGAGGGTTTTACTGTCTCTTACTTCCAATCAGTGAAATTGAC
CTTCCCGTGAAGAGGCGGGAATAAAAAAATAAGACGAGAAGACCCTATGGAGCTTTAATTAA
CTACTCCAAAAGTTAAACAATTCAACCACAAAGGGATAAAACATAACTTAACATGGACTAGCA
ATTTCGGTTGGGGTGACCTCGGAGTACAAAAAACCCTCCGAGTGATTTTAATCTAGACAAAC
CAGTCAAAATAACCATAACATCACTTATTGATCCAAAATTTTGATCAACGGAACAAGTTACCCT
AGGGATAACAGCGCAATCCTATTCTAGAGTTCCTATCGACAATAGGGTTTACGACCTCGATGT

TGGATCAGGACACCCAAATGGTGCAACCGCTATTAAAGGTTCGTTTGTTCAACGATTAAAGT
CCTACGTGATCTGAGTTCAGACCGGAGCAATCCAGGTCGGTTTCTATCTATTATAAATTTCTC
CCAGTACGAAAGGACAAGAGAAATGGGACCAACCTCACAAACGCGTCTCAGAGATAATTAAT
GATATAATCTTAACCTAATTAACTCATAATAAATCCAGCCCTAGAACAGGGCACATTAGGGTGG
CAGAGACCGGTAATTGCGTAAAACTTAAACCTTTATTACCAGAGGTTCAACTCCTCTCCCTAA
TAGCATGTTCATAATTAACATTCTAAGCCTAATCATTCCTATCCTACTGGCCGTAGCATTCCTC
ACCCTAGTAGAACGAAAAGTACTAGGTTATATGCAACTACGAAAAGGACCCAACGTTGTAGG
CCCCTACGGCCTACTCCAACCCATCGCCGATGCCCTAAAACTATTCACCAAAGAACCCCTAC
GACCAGCCACATCCTCAATCTCCATGTTCATTATTGCACCAATCCTAGCCTTATCCCTAGCAC
TAACAATATGAGTTCCACTACCAATACCCTACCCTCTAATCAACATAAATCTAGGAGTACTATT
CATGCTAGCCATGTCAAGCCTAGCAGTCTATTCTATCCTATGATCAGGATGAGCATCTAACTC
AAAATACGCACTCATCGGGGCCCTACGAGCAGTAGCCCAAACAATTTCATATGAAGTAACAC
TGGCAATCATCCTACTATCAGTGCTCCTAATAAATGGATCATATACTCTATCCACCCTAATCAC
AACACAAGAGCACATTTGAATAATCTTTACATCCTGACCCCTAGCCATAATATGATTTATCTCA
ACCCTAGCAGAAACCAACCGAGCCCCGTTCGACCTTACAGAAGGAGAGTCAGAACTTGTAT
CAGGCTTTAACGTAGAATATGCAGCCGGACCTTTCGCCATATTCTTCATAGCAGAATATGCCA
ACATCATCATAATAAATGCATTCACAGCAATTCTCTTCCTAGGAGCATTCCACGACCCACACA
CATCAGAACTATATACAATCAACTTCGTACTAAAAACACTCGCATTAACAATCACCTTCCTATG
AATCCGAGCATCATACCCACGATTCCGATATGACCAACTAATACATTTACTATGAAAAAGCTTC
CTGCCCCTAACACTAGCTCTATGTATATGACACATCTCACTCCCTATTATAACAGCAAGTATTC
CCCCACAATCATAGAAATATGTCTGATAAAAGAGTTACTTTGATAGAGTAAAAAATAGAGGTTC
AAACCCTCTTATTTCTAGAACAATAGGACTCGAACCTAAACCTGAGAATTCAAAATTCTCCGT
GCTACCAAAATACACCACATTCTACAGTAAGGTCAGCTAAGCTAAGCTATCGGGCCCATACC
CCGAAAATGTTGGTTCATACCCTTCCCATACTAATAAATCCCATTATCTACACTACCCTTATCAT
AACAGTAATATCCGGAACCATACTAGTAATAATCAGCTCACACTGACTACTCATCTGAATCGG
ATTCGAAATAAACCTATTAGCAATAATCCCAGTATTAATAAAAAATTTTAACCCACGAGCCACA
GAAGCAGCCACAAAATATTTCCTAACACAAGCCACAGCCTCCATGATACTAATAATAGCCATC
ATCATCAACCTCCTATATTCTGGCCAATGGACCATTACAAAAATATTTAACCCAGTAGCAATAA
CAATAATAACCCTGGCCCTAGCCATAAAACTAGGACTCTCACCTTTCCACTTCTGAGTCCCA
GAAGTAACCCAAGGCATTTCACTACAAGCAGGCCTACTATTACTAACATGACAAAAACTAGCC
CCATTATCAGTACTATGCCAAATCTCACAATCAATCAACCCAAACCTAATATTAACTATGGCCA
TATTATCAATTTTAATCGGAGGGTGAGGGGGACTAAACCAAACCCAACTTCGAAAAATCATAG
CATATTCATCAATCGCACACATAGGATGAATGACAGCAGTATTACCATATAACACAACCATAAC
AATCTTAAACCTACTAATTTACATCACAACAACACTAGCAATATTCATACTATTAATCCACAGCT
CAGCAACCACAACTTTATCCCTATCCCATACATGAAACAAGATACCCATCATCACAAGCCTAA
TAATAGTAACCCTACTCTCAATAGGAGGCCTGCCTCCACTATCAGGATTTATACCAAAATGAAT
AATCATTCAAGAAATAACAAAAAATGAAAGCATCATCATGCCAACACTCATAGCAATAACAGC
ACTGCTAAACCTCTATTTCTACATACGACTAGCCTACTCCTCCTCACTGACTATGTTCCCATC
CACCAATAACATAAAAATAAAATGACAATTCGAACACACAAAACAAATAAAACTACTTCCCACA
ATAATTGTATTATCGACACTAATCCTACCTATAACACCAGCCCTCTCGTCCCTAAACTAGGAAT
TTAGGTTAACACAGACCAAGAGCCTTCAAAGCTCTAAGTAAGTACAAAGTACTTAACTCCTGA
AAACCTAAGGACTGCAGGATTCATCCTACATCAATTGAATGCAAATCAAACACTTTAATTAAG
CTAAATCCTCACTAGATTGGTGGGATTACATACCCACGAAACTTTTAGTTAACAGCTAAACAC
CCTAATCAACTGGCTTCAATCTACTTCTCCCGCCGCAGGAAAAAAAAGGCGGGAGAAGTCC
CGGCAGAATTGAAGCTGCTTCTTTGAATTTGCAATTCAACATGACATTCACCACGGAACTGG
CAAAAAGAGGGCTTAACCTCTGTCTTTAGATTTACAGTCTAATGCTTACTCAGCCATTTTACC
TATGTTCGTAAATCGTTGACTATACTCAACAAACCACAAAGACATCGGCACCCTGTACCTACT

ATTTGGTGCCTGAGCAGGAATAGTGGGCACTGCCTTGAGCCTACTAATTCGCGCTGAACTA
GGTCAGCCCGGAACCCTACTTGGCGATGATCAAATCTATAATGTAATTGTTACAGCTCATGCC
TTTGTAATAATCTTCTTTATAGTAATACCCATTATGATTGGGGGTTTTGGTAACTGACTCGTACC
GCTAATAATCGGAGCTCCCGATATGGCCTTTCCACGTATAAACAACATAAGTTTCTGACTACT
TCCACCATCCTTCCTATTACTACTGGCATCCTCAATAGTAGAAGCCGGGGCGGGTACTGGAT
GAACCGTATACCCACCTTTAGCTGGAAACTTAGCCCATGCAGGAGCTTCAGTTGATCTAACA
ATTTTCTCCCTACACCTTGCAGGTGTATCATCAATCCTAGGGGCTATTAATTTCATTACCACAA
TTATTAACATAAAACCTCCCGCAATGTCTCAATACCAAACACCCCTGTTTGTCTGATCAGTAC
TAATCACAGCCGTACTACTTCTACTATCCCTGCCAGTTCTAGCAGCTGGCATTACTATACTACT
GACAGACCGCAACCTGAACACAACCTTTTTTGATCCAGCAGGTGGTGGAGACCCTATCCTT
TATCAACACTTGTTCTGATTTTTCGGACACCCAGAAGTATATATTCTCATCTTACCAGGGTTCG
GAATAATCTCCCACATTGTAACCTACTATTCAGGTAAAAAGAACCATTTGGATATATAGGCAT
AGTATGAGCCATAATGTCCATTGGATTCTTAGGTTTTATCGTATGGGCTCACCACATATTCACC
GTAGGAATAGACGTGGATACCCGAGCATACTTTACATCTGCCACAATAATCATTGCTATTCCC
ACTGGAGTAAAAGTATTTAGTTGATTAGCTACCCTGCACGGCGGCAATATTAAATGATCACCC
GCAATACTATGAGCTCTGGGCTTCATCTTCCTATTCACCGTAGGAGGTCTAACGGGCATTGT
ACTAGCTAACTCCTCCCTAGACATTGTATTACATGATACATATTATGTAGTCGCACACTTCCAC
TATGTCTTATCTATAGGAGCAGTGTTTGCCATTATAGGGGGCTTTGTTCACTGATTCCCCCTAT
TCTCCGGGTACACACTCAACCAAGCATGAGCAAAAATTCACTTTGTAATTATATTCGTAGGAG
TAAATATAACATTCTTTCCACAACACTTTCTAGGACTATCCGGAATACCTCGACGATACTCCGA
TTATCCTGACGCATACACAGCATGAAATACTATTTCCTCAATAGGCTCATTCATCTCACTAACA
GCAGTGATATTAATAATCTTCATTATCTGAGAAGCATTTGCATCAAAACGAGAAGTATCTGCAG
TAGAACTGACAAGCACAAACCTAGAATGACTACACGGATGTCCTCCTCCCTATCACACATTT
GAAGAACCAACATATATCAACCTAAAATAAGCATAAGAAAGGAAGGAATCGAACCCTCTCCCA
CTGGTTTCAAGCCAACGTCATAACCACTATGTCTTTCTCGATAATCGAGGTATTAGTAAAATAT
TACATAACTTTGTCGAAGTTATATTATAGGTGAAAGCCCTATATGCCTCTATGGCTTACCCTTT
CCAACTAGGCTTCCAAGACGCCACTTCACCCATCATAGAAGAACTCCTACACTTTCACGATC
ACACCTTAATAATTGTATTCTTAATCAGCTCTTTAGTGTTATATATCATTTCACTTATACTAACAA
CAAAACTGACACACACTAGCACAATGGATGCCCAAGAAGTAGAAACAATTTGAACAATCCTA
CCCGCTATTATTTTAATTCTTATTGCCCTTCCATCATTACGAATCCTTTATATAATAGACGAAATT
AATAACCCAGCCTTAACCGTAAAAACCATAGGACATCAATGATACTGAAGCTATGAGTATACA
GACTATGAAGACCTCACCTTTGACTCATATATAATCCCCACATCAGATCTTAAACCTGGAGAA
ATACGACTACTAGAAGTAGACAATCGAGTTGTTCTGCCAATAGAAATAACAATCCGAATATTAG
TGTCCTCTGAAGACGTACTACACTCATGAGCTGTCCCATCCCTCGGTTTAAAAACAGATGCT
ATCCCAGGACGACTAAACCAAACAACTCTAATATCCACACGACCTGGCCTTTATTACGGACA
GTGCTCAGAAATCTGTGGATCAAACCACAGCTTCATGCCCATTGTACTTGAACTTGTCCCAT
TAAAGTACTTCGAAAAATGGTCAACATCAATATTAACAGGTTCATTGAGAAGCTAGTCAGCAC
TAACCTTTTAAGTTAGAGATCGGGAGCCTAAATCTCCCCTCAATGGTATGCCACAACTAGATA
CATCTACATGATTCATTACAATTACATCAATAATTATAACATTATTTATTTTATTCCAACTAAAAAT
CTCAAACTACTCATACCCAGCAAGCCCAGAATCAACCGAACTCAAAACTCAAAAACATAGCA
CCCCTTGAGAAATAAAATGAACGAAAATCTATTTGCCTCTTTCATTGCCCCTACGATAATAGG
ACTACCTATTGTCACCTTAATTATTATATTCCCAAGCTTACTATTCCCAACACCCAAACGACTC
ATTAATAACCGCACAATCTCGATCCAACAATGATTAATCCAACTAACATCCAAACAAATAATGG
CTATTCACAACCAAAAAGGCCAAACCTGATCACTAATACTTATATCTCTAATTATATTCATTGGC
TCAACAAACATCCTAGGCCTACTACCACACTCATTCACACCCACCACACAACTATCAATAAAC
CTGGGTATAGCAATCCCCCTATGATCAGCAACCGTATTCACAGGATTCCGCCATAAAACCAA
AACATCACTAGCCCACTTTCTACCACAAGGAACACCCGCCCCATTAATTCCTATGCTCGTAAT

TATTGAAACTATTAGCCTATTTATTCAACCAGTAGCCCTAGCCGTACGACTGACAGCCAACAT
TACAGCAGGGCACCTATTAATTCATCTAATTGGAGGGGCCACATTAGCACTACTCAACATCAG
CACTATAACAGCTTTTATCACATTTACTATCCTCATCCTATTAACTATTCTTGAATTTGCAGTAG
CTCTGATCCAAGCTTATGTGTTTACACTGCTAGTAAGCTTATACCTACACGACAATACATAATG
ACCCACCAAACACATGCATACCACATAGTAAACCCAAGCCCATGACCACTTACCGGAGCCCT
ATCAGCCCTTTTAATAACATCAGGCCTAATTATATGATTCCACTTTAACTCTATACTCTTACTAT
CTCTAGGACTATTAACCAATACTTTGACAATATACCAATGGTGACGAGACATTATTCGAGAAA
GCACTTTCCAAGGCCACCACACATCAGTCGTCCAAAAAGGCTTACGATACGGTATAATTTTAT
TTATTATTTCCGAGGTTCTGTTCTTCACTGGATTCTTTTGAGCTTTCTACCACTCAAGCCTAG
CACCAACACCCGAATTAGGAGGTTGCTGACCACCAACAGGAATTCACCCACTAAACCCCCT
AGAAGTACCCCTACTAAACACCTCAATCCTCCTCGCCTCAGGAGTATCCATTACCTGAGCCC
ATCACAGCCTAATAGAAGGGGACCGAAAACACATAATCCAAGCACTATCCATCACCATTGCA
CTAGGCGTATACTTCACCCTCCTCCAAGCCTCAGAATATTACGAAGCACCATTCACAATCTCC
GACGGAGTGTATGGATCCACTTTCTTTGTGGCTACAGGATTTCACGGGTTGCACGTAATCAT
CGGATCTACTTTCCTAGCAGTGTGCTTACTACGACAACTAAAATTCCACTTCACATCCAACCA
CCACTTCGGCTTTGAAGCCGCAGCCTGATACTGACACTTCGTAGATGTAGTTTGACTATTCC
TTTACGTATCAATCTATTGATGAGGATCCTACTCTTTTAGTATTAAGCAGTACAATTGACTTCCA
ATCAATCAGTTTCGGTAAACTCCGAAAAAGAGTAATAAATATTATACTAACACTATTCACAAAC
GTAACCCTAGCCTCCCTACTCGTACTAATCGCATTCTGACTACCCCAACTAAACACATATTCA
GAAAAAACAAGCCCATATGAATGTGGATTTGACCCCATAGGATCAGCACGCCTCCCATTCTC
AATAAAATTTTTCCTAGTAGCCATTACATTTCTCCTTTTTGATCTAGAAATCGCCCTCCTCCTT
CCCCTACCATGAGCATCCCAAACAAACAATCTAAAAACAATACTTACAATAGCACTATTCCTTC
TTACCCTACTAGCAGCAAGCCTAGCATACGAATGAACCCAAAAAGGCCTAGAATGAACAGAA
TATGATAATTAGTTTAAAACAAAACAAATGATTTCGACTCATTAGACTATGATTTACTTCATAATT
ATCAAGTGCCATTAGTATACATAAACATCATTATAGCATTCACGATTGCCCTTGCAGGGTTACT
TATATATCGATCTCACTTAATATCTTCACTACTATGCCTAGAAGGAATGATATTATCACTATTCAT
CATATCGACTCTAATTGTCCTAAACACACACTTCACCCTAGCTAACATAATACCCATTATTTTAC
TAGTGTTTGCAGCCTGCGAAGCTGCACTGGGCCTATCACTACTAGTAATAGTATCCAACACA
TACGGTACCGATTACGTCCAAAACTTAAACCTCTTACAATGCTAAAAATTATTATCCCAACAAC
AATACTACTACCCATAACATGAATATCTAAACACAACATAATCTGAATCAATGCAACAGTACATA
GTCTCCTCATTAGCCTGATCAGTCTATCCCTACTAAACCAACTAGGCGAAAACAGCCTTAATT
TTTCCTTAACATTCTTCTCCGACTCACTATCAGCACCCCTACTAGTTCTAACCACATGACTCC
TCCCCCTTATACTAATAGCTAGCCAATCCCACCTATCAAAAGAAACCACAACCCGAAAAAAAC
TATATATTACCATACTAATCCTACTACAACTATTCCTAATTATAACCTTCACCGCCACCGAACTA
ATCTTATTCTATATCCTATTCGAAGCAACACTAGTACCCACACTAATCATCATCACACGCTGAG
GAAACCAAACAGAACGACTCAATGCAGGACTTTATTTCCTATTCTACACCCTAGCAGGATCC
CTACCACTGCTAGTAGCACTAGTTTATATCCAAAACACCACAGGCTCACTAAACTTCTTAATTA
TCCATTACTGATCCCACCCATTATCCAACTCTTGATCAAACATTTTTATATGATTAGCATGCATC
ATAGCCTTCATAGTAAAAATACCTCTATACGGACTCCATCTTTGACTGCCAAAAGCCCATGTA
GAAGCCCCTATTGCAGGTTCAATAGTACTTGCAGCCGTACTGCTAAAACTCGGAGGCTATGG
CATAATGCGAATCACTACTATTCTAAACCCACTAACAAACTACATAGCCTATCCATTCCTCATG
CTTTCCATATGAGGTATAATCATAACCAGCTCTATCTGCTTACGTCAAACCGACCTAAAATCCT
TAATCGCTTATTCATCAGTAAGTCATATAGCACTTGTAATCGTAGCAATCATAATTCAAACCCC
CTGAAGCTTCATAGGAGCCACAGCTCTCATAATTGCCCACGGACTAACATCCTCCATACTATT
CTGCCTAGCCAACACTAACTATGAACGAGTACACAGCCGAACCATAATCCTAGCCCGAGGA
CTGCAAACACTCCTACCACTCATAGCAACATGATGACTAATAGCAAGCCTCACAAACCTAGC
CCTACCCCCATCCATCAATCTAATCGGAGAATTATTTATCATTACAGCATCATTTTCATGATCCA

ACATCACAATTATTCTCATAGGAATAAACATAATAATTACAGCTCTCTACTCTCTCTACATACTA
ATTATTACACAACGAGGAAAATACACCCACCACATTAACAACATCAAACCCTCATTCACACGA
GAAAACGCCCTCATAGCCCTACATATTCTACCACTACTACTACTGACCTTAAACCCTAAAATAA
TCCTAGGACCCCTTTACTGTAGATATAGTTTAATAAAAACCCTAGATTGTGAATCTAGTAATAG
AAAATTAAATATTCTTATCTACCGAAAAAGTTTGCAAGAACTGCTAACTCATGCTTCCACACTT
AAAAATGTGGCTTTTTCAACTTTTAAAGGATAGCAGTTATCCGTTGGTCTTAGGAACCAAAAA
ATTGGTGCAACTCCAAATAAAAGTAATAAACCCATTCGCCTCACTCACATTAACCACACTGAC
TATTCTAACCATCCCAATTATAATATCCAACTCAAACATCTACAAAACTAACCTTTACCCTAACT
ACGTAAAAACCACCGTATCCTACGCCTTCACTCTCAGCCTAGTCCCCTTACTAATATTTATACA
CACAGGCCAAGAAATAATCATTTCAAACTGACATTGAATAACCCTACAGACCGTAGAACTCTC
TCTTAGCTTTAAAATAGACTATTTCTCAGTAATATTCATTCCCGTAGCACTATTCGTCACATGAT
CAATTATAGAATTCTCCATATGATACATACACTCAGACCCCTTCATCAACCGATTCTTTAAATAC
CTACTACTATTCTTAATCACTATAATAATCCTCGTAACCGCCAACAACCTCTTCCAACTCTTTAT
CGGATGAGAAGGCGTAGGAATCATATCATTCCTCCTAATCGGATGATGACACGGACGAACAG
ACGCCAACACAGCTGCACTACAAGCAATCCTATACAACCGCATCGGAGACATTGGATTTGTC
CTATCCATAGCATGATTCTTAACCCACTCAAACGCATGAGATTTTCAACAAATCTTTATACTAA
ACAATGAATGCCCAAACATACCATTAATCGGCCTACTCCTAGCTGCAGCAGGAAAATCAGCT
CAATTCGGACTACATCCCTGATTGCCCTCAGCAATAGAAGGCCCAACTCCCGTATCAGCATT
ACTACACTCCAGTACAATAGTAGTAGCAGGGGTATTTCTACTCATCCGCTTCTACCCCTTAAT
AGAAACTAACAAACTAGTTCAAACCATAACACTATGCCTAGGAGCTATCACCACCTTATTTAC
AGCACTATGTGCAATCACACAAAATGATATCAAAAAAATCGTAGCCTTCTCAACTTCAAGCCA
ACTAGGCTTGATAATAGTGACAATCGGCATCAACCAACCCCACCTAGCATTTCTTCATATCTG
CATGCACGCTTTCTTCAAAGCAATACTATTCATATGCTCCGGATCCATTATCCACAGCCTCAAT
GACGAACAAGACATCCGAAAAATAGGCGGACTGTATAAAGCAATACCATTCACAACAACAGC
ACTAATTATTGGAAGCCTGGCATTAACAGGAATGCCTTATCTCACAGGATTCTACTCAAAAGA
CCTTATCATTGAAGCAGCAAACATATCCTACACAAACGCCTGAGCCCTACTAATAACATTAATT
GCCACATCCCTAACCGCTGCCTACAGCACTCGAATTATCTTCTTTGCATTCCTAGGGCAACC
ACGTTTCCCACCCCTAGTCCTAATTAATGAAAATAACCCCCTACTAATTAACTCTATTAAACGC
CTTTTAATCGGAAGCATCTTCGCTGGCTTTATCATCTCCAACAACATCCCACCAATAACAGTA
CCAAACACAACAATACCCCTTTACATAAAAATAACAGCCCTAATCGTAACCATCATAGGATTCA
TACTAGCCCTAGAGCTAAACAACACAACCTACTACCTGAAATTTAAATACCCATCACAAACATA
CAAATTTTCCAACATACTAGGATATTATCCCTCCATCATACACCGCCTACCAACATACCACAAC
CTGTCTATAAGCCAAAAATCCGCATCATCATTACTAGACTTAATTTGACTAGAAACTATTCTAC
CAAAAACAACCTCTTTCATCCAAATAAAAATATCAATTATAGTATCAAATCAAAAAGGCCTAATC
AAACTATACTTTCTCTCCTTCCTAATCACTATTATAATCAGCATAATACTATTTAATTACCACGAG
TAATCTCCATAATAACAACAACTCCAATAAGCAATGATCAACCAGTAACAATAACTAATCAAGT
ACCATAACTATATAAAGCAGCAATCCCCATAGCTTCCTCACTAAAAAACCCTGAATCACCCGT
ATCATAAATTACTCAATCCCCAAGCCCATTAAACTTAAAAATAATTTCTACTTCCTCTTCCTTCA
ATGCATAATAAACCATACAAAACTCCATTATTAAACCAGAAACAAATGCTCCAAAAACAGTCTT
ATTAGAAACTCAAACCTCAGGGTACATCTCAGTAGCCATGGCAGTAGTATAACCAAAAACCA
CTAACATACCCCCCAAATAAATCAAAAACACCATTAAACCTAAAAAAGACCCACCAAAATTCA
ATACAATACCACAACCAACTCCACCACTTACAATCAACCCAAGTCCACCATAAATAGGAGAG
GGTTTAGAAGAAAAACCAACAAACCCAATAACAAAAATAGTACTTAAAATAAATGCAATATATAT
TGTCATTATTCTCACATGGAATCTAACCACGACCAATGACATGAAAAATCATCGTTGTACTTCA
ACTACAAGAACCTTAATGACCAACATCCGAAAATCACACCCACTAATAAAAATTATCAACAAC
GCATTCATTGACCTCCCAGCCCCCTCAAACATCTCATCATGATGAAACTTCGGTTCCCTCTTA
GGCATCTGCCTAATCTTGCAAATCCTAACAGGCCTGTTCTTAGCAATACATTACACATCAGAC

ACAACAACAGCTTTCTCATCAGTTACACACATCTGTCGAGACGTAAATTACGGATGAGTTATT
CGCTACCTACATGCAAACGGAGCATCCATGTTCTTTATTTGCCTATTCATCCACGTAGGCCGA
GGCCTATACTACGGATCCTATATATTCCTAGAAACATGAAACATTGGAGTAGTCCTACTATTTA
CCGTTATAGCAACAGCCTTCATAGGCTACGTCCTGCCCTGAGGACAAATATCATTCTGAGGA
GCTACGGTCATCACAAATCTACTATCAGCTATCCCTTATATCGGAACAGACCTCGTAGAATGA
ATCTGAGGGGGCTTTTCCGTCGACAAAGCAACCCTCACACGATTCTTCGCCTTTCACTTTAT
CCTGCCATTCATCATTACCGCCCTCGCAGCCGTACATCTCCTATTCCTGCACGAAACCGGAT
CCAACAACCCTACCGGAATCTCATCAGACATAGACAAAATTCCATTTCACCCATACTACACTA
TTAAAGACATTCTAGGGGCCTTATTTATAATACTAATCCTACTAATCCTTGTACTATTCTCACCA
GACCTACTAGGAGACCCAGACAACTACACCCCAGCAAACCCACTAAACACCCCACCCCATA
TTAAACCAGAATGATATTTCTTATTCGCCTACGCTATCCTACGTTCAATTCCTAATAAACTAGGT
GGAGTGCTAGCTCTAATAGCCTCCATCCTAATCCTAATTTTAATGCCCATACTACACACATCCA
AACAACGAAGCATAATATTTCGACCACTAAGTCAATGCCTATTCTGAATACTAGTAGCAGACC
TCATTACACTAACATGAATTGGAGGACAACCCGTAGAACACCCATTCATCATCATCGGCCAA
CTAGCCTCCATCTTATATTTCCTAATCATTCTAGTATTGATACCAATCACTAGCATCATCGAAAA
CAACCTATTAAAGTGAAGAGTCTTTGTAGTATATAAAATACCCTGGTCTTGTAAACCAGAAAA
GGAGGACCACCCCTCCCCAAGACTCAAGGAAGGAGACTAACTCCGCCATCAGCACCCAAA
GCTGAAATTCTAACTAAACTATTCCCTGCAACCAAAACAAGCATTCCATTCGTATGCAAACCA
AAACGCCAAGTACTTAATTACTATCTTTAAAACAAAAAAACCCATAAAAATTGCGCACAAACAT
ACAAATATGCGACCCCAAAAATTTAACCATTAAAAACAAAAAATTTAATATATTATAGCCCTATG
TACGTCGTGCATTAACTGCTAGTCCCCATGCATATAAGCATGTACATATTATTATTAATATTACAT
AGTACATATTATTATTGATCGTACATAGCACATATCATGTCAAATAACTCCAGTCAACATGCGTA
TCACCACCATTAGATCACGAGCTTAATTACCATGCCGCGTGAAACCAGCAACCCGCTTGGCA
GGGATCCCTCTTCTCGCTCCGGGCCCATAAATCGTGGGGGTTTCTACTGATGAACTTTAACA
GGCATCTGGTTCTTACTTCAGGACCATCTCATCTAAAATCGCCCACTCTTTCCCCTTAAATAA
GACATCTCGATGGACTAATGACTAATCAGCCCATGCTCACACATAACTGAGGTTTCATACATT
TGGTATTTTTTAATTTTTGGGGATGCTTGGACTCAGCCATGGCCGTCAAAGGCCCTAACACA
GTCAAATCAATTGTAGCTGGACTTCATGGAACTCATGATCCGGCACGACAATCCAAACAAGG
TGCTATTCAGTCAATGGTTACGGGACATAACGTGCGTACACGTGCGTACACGTGCGTACAC
GTGCGTACACGTGCGTACACGTGCGTACACGTGCGTACACGTGCGTACACGTGCGTACAC
GTGCGTACACG
