# Supplementary material for: Characterization of the Mitochondrial Genome of the Vietnamese Central Highland Wild Boar (Sus scrofa)
Source: Animals (Basel). 2025 Jul 10;15(14):2029. doi: 10.3390/ani15142029 (PMC12291927; doi:10.3390/ani15142029)
Supplement: Supplementary file 1 [file animals-15-02029-s001.zip › Table S2.pdf]

Supplementary Table S2: Read quality (Illumina) of analyzed samples.

Sample		Total reads	Total bases	Read length (bp)	%GC	%Q30
Pre-purification	Read R1	21,060,434	3,159,065,100	35-151	44.3	95.9
	Read R2	21,060,434	3,159,065,100	35-151	44.4	95.4
Post-purification	Read R1	20,949,602	2,825,893,364	35-151	43.5	96.1
	Read R2	20,949,602	2,825,893,364	35-151	43.5	96.1
