# Supplementary material for: Characterization of the Mitochondrial Genome of the Vietnamese Central Highland Wild Boar (Sus scrofa)
Source: Animals (Basel). 2025 Jul 10;15(14):2029. doi: 10.3390/ani15142029 (PMC12291927; doi:10.3390/ani15142029)
Supplement: Supplementary file 1 [file animals-15-02029-s001.zip › Table S3.pdf]

Supplementary Table S3: De novo assembly result

Number of contigs	1
Number of loops	1
%GC	39.4
Total length of contigs (bp)	16,581
Mean coverage of read assembly	63.3178
